# Repair of high-flow cerebrospinal fluid leak by combined artificial dura plug and free mucosal flap in 15 cases

**DOI:** 10.3389/fsurg.2025.1422524

**Published:** 2025-04-22

**Authors:** Hengsen Zhang, Duoduo Li, Zhitong Liu, Bin Chen, Pasut Limchoopornwikul, Yibao Wang, Yong Wang

**Affiliations:** ^1^ Department of Neurosurgery, The First Affiliated Hospital of China Medical University, Shenyang, China; ^2^ Department of Neurosurgery, Affiliated Hospital of Jiangnan University, Wuxi, China; ^3^ Wuxi School of Medicine, Jiangnan University, Wuxi, China; ^4^ Department of Neurosurgery, Hamamatsu University School of Medicine, Hamamatsu, Japan; ^5^ Department of Neurosurgery, Chongqing General Hospital, Chongqing University, Chongqing, China

**Keywords:** cerebrospinal fluid leak, endoscopic endonasal approach, free mucosal flap, skull base, reconstruction

## Abstract

**Background:**

In the endoscopic endonasal approach skull base surgery repair and reconstruction of the base of the skull is a critical step. Free mucosal flaps are primarily used to repair low-flow cerebrospinal fluid (CSF) leaks, whereas they are not adequate in the face of high-flow CSF leaks. We propose a modified approach-termed the “Fishing method”-which utilizes free mucosal flaps in combination with absorbable sutures and an artificial dura to reverse-plug the defect, to repair high-flow CSF leaks with a clear point of origin.

**Objective:**

To investigate the application of the “Fishing method” to repair high-flow CSF leaks caused by large diaphragma sellae rupture and small dura leak that occur unexpectedly during endoscopic endonasal sellae area surgery.

**Methods:**

A retrospective analysis was conducted including 15 patients with unexpected intraoperative high-flow cerebrospinal fluid leaks. The “Fishing method” was applied to reconstruct and repair the skull base in these patients, and the results were evaluated.

**Results:**

In 10 cases of large diaphragma sellae rupture that occurred during pituitary adenomas resection, all 10 patients were successfully repaired in a single operation using the “Fishing method”, with no cerebrospinal fluid nasal leakage (100%); in 5 cases of small dura ruptures that occurred during chordoma resection, 4 patients underwent successful repair in a single operation, with no cerebrospinal fluid nasal leakage occurring in 80% of cases, resulting in an overall success rate of 93.3%.

**Conclusion:**

The “Fishing method” is a reliable technique for skull base reconstruction and serves as an effective solution for high-flow CSF leaks caused by unexpected large diaphragma sellae rupture or a small dura leak occurring intraoperatively.

## Introduction

With the continuous advancement of endoscopic skull base surgery, the endoscopic endonasal approach (EEA) has become the preferred surgical technique for the management of lesions located near the midline of the skull base. However, effectively addressing cerebrospinal fluid (CSF) nasal leakage, the most common complication of this procedure, is crucial for ensuring the overall success of the surgical treatment. Building on the free mucosal flap technique for repairing CSF leaks (1), we propose an enhanced method for managing large diaphragma sellae ruptures or small dura leaks that occur unexpectedly during surgery. This approach combines the use of an artificial dura counter plug (“Fishing method”) with a free mucosal flap. Between November 2017 and March 2021, we applied this technique to repair high-flow CSF leaks caused by intraoperative diaphragma sellae rupture or dura leak in 15 patients, achieving a success rate of 93.33%. This method is straightforward, easy to implement, and relies on readily available materials, making it highly suitable for widespread clinical adoption.

## Methods

Between November 2017 and March 2021, fifteen consecutive patients (seven males, eight females; mean age, 53 years) with high-flow CSF leakage caused by diaphragma sellae rupture or dura leak during the endoscopic surgery were included in this study. All patients had a confirmed diagnosis of tumors in the sellae region, including 10 cases of pituitary adenomas and 5 cases of chordomas.

Informed consent for the endoscopic procedure was obtained from all patients, and the institutional review board approved the retrospective review of these cases. During surgery, under general anesthesia, the diaphragma sellae or dura was occasionally torn due to tight adhesions between the tumor and these structures during tumor removal. In cases of intraoperative diaphragma sellae rupture or dura leak (defects up to 3 mm in diameter), the defect was initially blocked with a small cotton piece to temporarily control CSF leakage. Definitive repair was performed after the tumor resection. For the repair, depending on the size of the defect, an artificial dura (Tech Thinkful, 1350–2013) patch measuring 2 to 3 times the size of the defect was prepared. A 4–0 absorbable suture was passed through the center of the patch and tied, leaving an adequate free end. The surgeon then used a suction device with a scraping spoon to guide the artificial dura into the upper portion of the diaphragma sellae or dura defect. The free end of the suture was gently pulled in the opposite direction, embedding the patch into the defect in a manner resembling a bathtub plug. The degree of insertion and blockage is appropriate to significantly reduce cerebrospinal fluid leakage and to avoid further tearing of the sellae septum or dura mater due to excessive traction. This process is similar to fishing reeling action, so we also call it the “Fishing method”.

**Figure 1:**
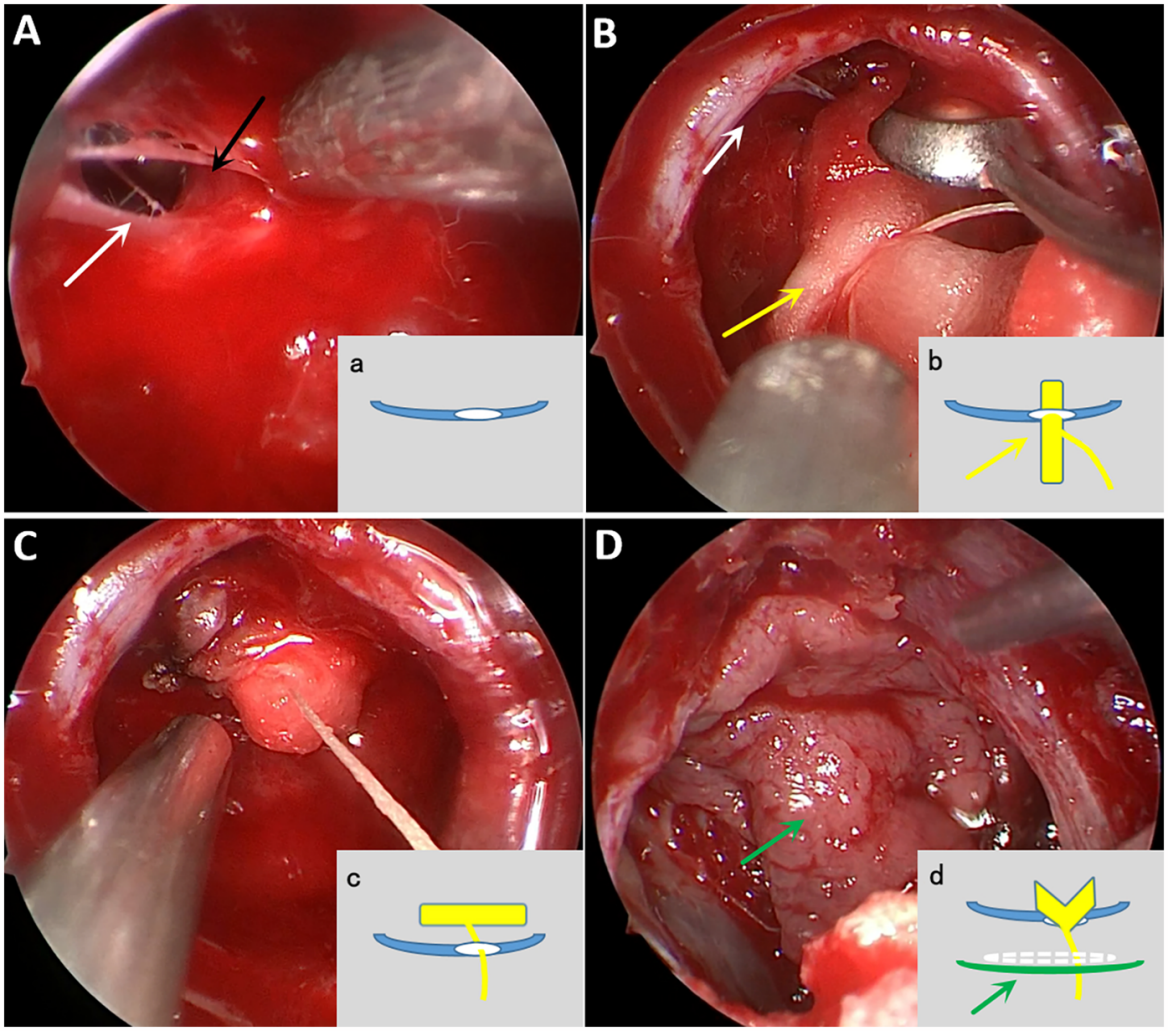
Intraoperative screenshot and abbreviated schematic of “Fishing method” procedure. **(A)** Though the large diaphragma sellae defect (white arrow), the pituitary stalk (black arrow) can be seen. Schematic a, blue represents the diaphragma sellae and white represents the diaphragma sellae defect. **(B)** Gently insert the artificial dura (yellow arrow) with absorbable suture all the way into the diaphragma sellae defect. Schematic b, yellow rectangles in the schematic represent the artificial dura and yellow solid lines represent the absorbable threads. **(C)** Pulling the absorbable suture so that the artificial dura fills the defect from top to bottom. **(D)** The sellae can be properly filled with artificial dura again, followed by uniform and tight application of free middle turbinate mucosal flap (green arrow) at the level of the sellae floor, and then reinforced with bioprotein gel spray. Schematic d, white dashed line is artificial dura filling, green solid line represents free middle turbinate mucosa.

To further secure the repair, bioprotein glue was sprayed on and around the defect site. The sellae cavity was filled with a combination of artificial dura and gelatin sponge, and the repair was reinforced with free mucosal flaps positioned evenly at the base of the sellae. Additional layers of bioprotein glue were applied to ensure a tight seal (Figure 1). Iodoform oil gauze (supplemented by a water-filled balloon if necessary) was placed in the sphenoid sinus cavity to provide additional support. The patients were able to breathe as the packing did not involve the posterior nostrils. The balloon was removed on postoperative day 7, while the iodoform gauze was removed after two weeks. All patients were able to mobilize on the first postoperative day, and routine lumbar drainage was not performed.

## Results

**Figure 2:**
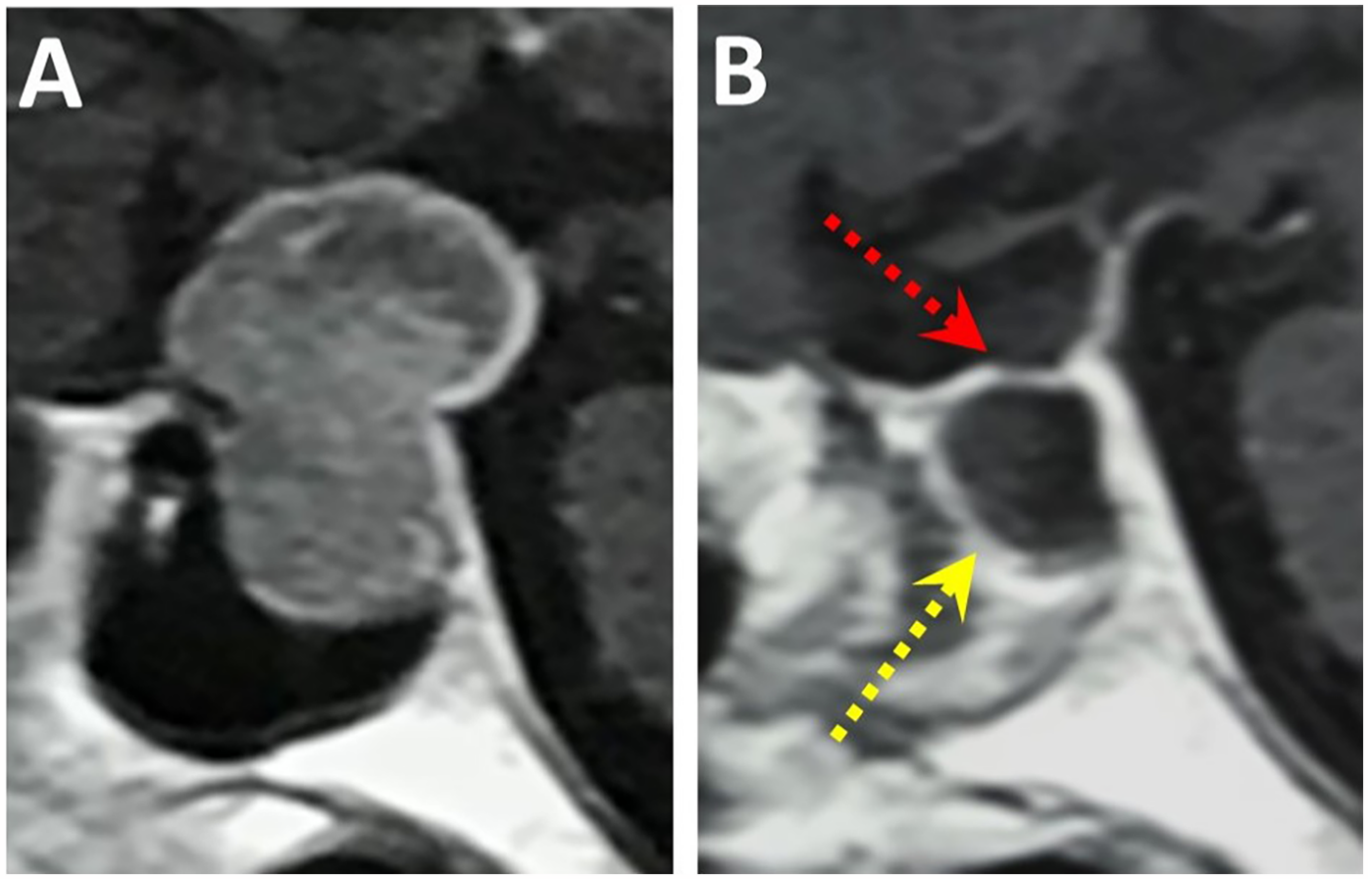
Pituitary tumor MRI before and after surgery **(A)**, preoperative T1-enhanced sagittal view showing pituitary tumor occupancy in the sellae area. **(B)** Postoperative review at 6 months showed good free mucosal growth (yellow arrow) and intact diaphragma sellae (red arrow).

Fifteen patients who experienced an unexpected defect in the diaphragma sellae or dura during surgery were treated using the fishing method. Fourteen of these patients recovered without symptoms of CSF nasal leakage and were successfully discharged two weeks postoperatively. A follow-up conducted over six months confirmed that all 14 patients remained free of CSF nasal leakage (Figure 2). One patient with recurrent chordoma developed a nasal CSF leak combined with pneumocephalus on the fifth postoperative day. This patient was successfully treated with secondary repair using thigh fat with broad fascia and water balloon support for 10 days, and was subsequently discharged.

## Discussion

CSF nasal leakage is one of the most common complications associated with endoscopic skull base surgery (2–4). The incidence of CSF nasal leakage during transsphenoidal endoscopic endonasal pituitary adenomas resection ranged from 0.1% to 17% (5, 6). For suprasellar lesions such as craniopharyngioma or tuberculum sellae meningiomas, the incidence of CSF nasal leakage is even higher, ranging from 1.6% to 33% (7, 8). In the case of chordomas, which predominantly invade epidural tissues with limited penetration into the inner dura and arachnoid layer, the risk of CSF leakage remains significant due to the large resection area and depth of dissection. If the CSF leak is not adequately repaired during these endoscopic procedures, serious complications may arise, including intracranial infection, pneumocephalus, hydrocephalus, and, in severe cases, death due to shock from intracranial infection (9, 10). Therefore, effective management and repair of CSF leaks are critical to ensuring the success of endoscopic skull base surgeries.

During endoscopic endonasal resection of sellar tumors, small diaphragma sellae leaks can typically be repaired with high success by covering defect with a free middle turbinate mucosal flap, supported locally (11, 12). This approach is effective because the combined structure of the diaphragma sellae and the sellae floor provides additional support to prevent CSF leakage, while the mucosal flap facilitated reconstruction of the sellae floor. However, repairing large diaphragma sellae rupture presents a significant challenge due to the impact of high-flow CSF leakage, which hinders the adhesion and healing of the free mucosal flap and often results in repair failure. To address this issue, we implemented the “Fishing method” to repair unexpected large diaphragma sellae rupture occurring intraoperatively. This method involves using an artificial dura counter plug to seal the defect, converting a high-flow CSF leak into a low-flow leak. This key feature prevents the high-flow CSF leak from disrupting the healing process of the mucosal flap. Artificial dura is an excellent repair material (13) and the “Fishing method” is simple to perform. It allows patient to mobilize freely after surgery. Among 10 patients treated for large diaphragma sellae rupture using this method, all were successfully discharged without any postoperative CSF nasal leakage. We also applied this method to five cases of small dura leak encountered during endoscopic endonasal resection of chordomas. Four of these cases were successfully repaired. In the fifth case, however, the patient developed a sudden increase in CSF leakage and pneumocephalus on the fifth postoperative day, as confirmed by cranial CT. Endoscopic exploration revealed that the artificial dura had prolapsed from the defect. A secondary repair was performed using multiple layers of reinforcement with autologous fat and broad fascia harvested from the thigh, supported by a water-filled balloon. The patient recovered successfully and was discharged after the second repair.

We believe the prolapse of the artificial dura plug may have been caused by a sudden rise in intracranial pressure triggered by sneezing or straining during defecation. To prevent such complications, patients should be advised to avoid activities that may cause abrupt increases in intracranial pressure following CSF leak repair using this method. Moreover, in the event of tamponade failure during repair with the Fishing method, the use of a vascular pedicled nasoseptal flap remains necessary to ensure successful closure (14).

For larger dural defects and high-flow CSF leaks, vascularized mucosal flaps are commonly used for skull base reconstruction, with a postoperative cerebrospinal fluid leak rate often below 10% (15–18). In comparison, while the “Fishing method” does not significantly reduce the postoperative CSF leak rate, it provides a timely and effective solution for repairing and reconstructing unexpected intraoperative leaks. Additionally, it avoids nasal discomfort and severe crusting during the healing process caused by nasal septal mucosal defects. This technique was primarily applied during endoscopic endonasal resections of chordomas with partial dura invasion and pituitary adenomas. Additionally, it shows potential for repairing CSF leaks encountered during expanded endoscopic endonasal approaches. In such cases, the artificial dura is used as a counter-occlusion to reduce high-flow CSF leakage, followed by repair using a vascular pedicled nasoseptal flap. Recently, we successfully employed the “fishing method” combined with a vascular pedicled nasoseptal flap to repair a CSF leak during the resection of an inter-sellae craniopharyngioma, achieving satisfactory results and good postoperative recovery (this case is not included in this paper). For CSF leaks encountered during the resection of tumors such as pituitary adenomas invading the third ventricle floor, craniopharyngiomas, tuberculum sellae meningiomas, and chordomas with extensive subdural invasion, we continue to recommend skull base reconstruction using bone fragments in combination with a vascular pedicled nasoseptal flap for optimal outcomes (19, 20).

## Conclusions

The “Fishing method” is a reliable technique for skull base reconstruction, offering an effective solution for high-flow CSF leaks caused by large diaphragma sellae rupture or small dura leak. This method is straightforward to perform, utilizes readily accessible materials, and has demonstrated excellent clinical outcomes, making it a practical and easily adoptable approach in clinical practice.

## Data Availability

The original contributions presented in the study are included in the article/Supplementary Material, further inquiries can be directed to the corresponding author.
